# Significantly different clinical features between hypertriglyceridemia and biliary acute pancreatitis: a retrospective study of 730 patients from a tertiary center

**DOI:** 10.1186/s12876-018-0821-z

**Published:** 2018-06-19

**Authors:** Xiaoyao Li, Lu Ke, Jie Dong, Bo Ye, Lei Meng, Wenjian Mao, Qi Yang, Weiqin Li, Jieshou Li

**Affiliations:** Surgical Intensive Care Unit (SICU), Department of General Surgery, Jinling Hospital, Medical School of Nanjing University, Nanjing, China

**Keywords:** Biliary acute pancreatitis, Hypertriglyceridemia acute pancreatitis, Acute kidney injury(AKI), Body Mass Index(BMI), Infected pancreatic necrosis

## Abstract

**Background:**

Unlike western world, gallstones and hypertriglyceridemia (HTG) are among the first two etiologies of acute pancreatitis (AP) in China. But yet, detailed differences in clinical features and outcomes between hypertriglyceridemia and biliary acute pancreatitis have not been well described.

**Methods:**

This retrospective study enrolled 730 acute pancreatitis patients from July 1, 2013 to October 1, 2016 in Jinling Hospital. The causes of the study patients were defined according to specific diagnostic criteria. The clinical features and outcomes of patients with hypertriglyceridemia acute pancreatitis (HTG-AP) and biliary acute pancreatitis (BAP) were compared in terms of general information, disease severity, laboratory data, system complications, local complications, and clinical outcome.

**Results:**

In the enrolled 730 AP patients, 305 (41.8%) were HTG-AP, and 425 (58.2%) were BAP. Compared to BAP, the HTG-AP patients were found to be younger, with higher body mass Index (BMI), and much higher proportion of diabetes, fatty liver and high fat diet. Besides that, HTG-AP patients had significantly higher C-reactive protein (CRP) (*p*<0.01) and creatinine (*p* = 0.031), together with more acute respiratory distress syndrome (ARDS) (*p* = 0.039), acute kidney injury (AKI) (*p*<0.001), deep venous thrombosis (*p* = 0.008) and multiple organ dysfunction syndrome (MODS) (*p* = 0.032) in systematic complications. As for local complications, HTG-AP patients had significantly less infected pancreatitis necrosis (*p* = 0.005). However, there was no difference in mortality, hospital duration and costs between the groups.

**Conclusion:**

HTG-AP patients were younger, more male, having high fat diet and with higher BMI compared to BAP patients. The prevalence of AKI/ARDS/DVT/MODS in HTG-AP patients was higher than BAP patients, while BAP patients had a greater possibility in development of infected pancreatitis necrosis (IPN). According to the multivariate analysis, only the complication of AKI was independently related with the etiology of HTG, however, BMI contributes to AKI, ARDS and DVT.

**Electronic supplementary material:**

The online version of this article (10.1186/s12876-018-0821-z) contains supplementary material, which is available to authorized users.

## Background

Acute pancreatitis (AP) is an acute inflammatory disease which is characterized by local pancreatic inflammation and consequently systemic inflammatory response. The imaging of AP manifests as pancreatic edema or necrosis involving the pancreas as well as peripancreatic tissues and even distant organs [1, 2]. In the western countries, the most common etiology of AP was gallstones, followed by alcohol abuse and hypertriglyceridemia [3, 4]. While the incidence of hypertriglyceridemia acute pancreatitis (HTG-AP) was much higher in China and has been increasing year by year according to the recent studies [5–10].

Previous studies well reported the clinical features of AP with different etiologies, rather than the detailed differences between HTG-AP and BAP. In this retrospective study from July 2013 to October 2016, we compared the clinical features, complications and outcomes between hypertriglyceridemia and biliary acute pancreatitis patients, as the two leading etiologies of acute pancreatitis in China.

## Methods

### Patient selection

This study retrospectively screened 999 AP cases admitted to the Surgical Intensive Care Unit (SICU), Department of General Surgery from July,1 2013 to October,1 2016, Jinling Hospital, Medical School of Nanjing University.

Patients who met the following criteria were excluded: (1) re-admission to the SICU; (2) traumatic, neoplastic, parathyroidal or other idiopathic pancreatitis; (3) younger than 18 years old. Eventually, 730 AP patients were enrolled in this study. The diagnosis and classification of the severity of AP were defined according to the 2012 revision of the Atlanta Classification.

### Data collection

The data analyzed in this study included general information as sex, age, body mass index (BMI), diabetes, fatty liver, high fat diet, transfer from other hospitals and clinical features as pancreatitis severity, incidence of systemic and local complications, mortality. Levels of hemoglobin, hematocrit, platelet, C-reactive protein (CRP), IL-6, creatinine, alanine aminotransferase (ALT) and other laboratory results were included in the comparison. All the laboratory results were obtained from the Central Laboratory of Jinling Hospital according to the standard protocols. Meanwhile, acute physiology and chronic health evaluation II (APACHE II) score was manually calculated for each single patient.

According to the 2012 revised of the Atlanta Classification the etiology of AP and the definition of complications, including portal vein thrombosis, intra-abdominal hypertension and hemorrhage, deep vein thrombosis (DVT) and gastrointestinal fistula, were judged by two independent physicians [1]. Different severity of AP as mild, moderate and severe, was assessed based on the presence of local or systemic complications and transient/persistent organ failure [2].

### Definition

The diagnosis of AP requires at least two of the following three features: (1) abdominal pain consistent with the disease (2) serum lipase activity (or amylase activity) at least three times greater than the upper limit of normal; and (3) characteristic findings from abdominal imaging [1].

The etiology of AP was analyzed by the following criteria. Biliary acute pancreatitis required the confirmation of gallstones or biliary sludge by any kind radiological imaging, including endoscopic ultrasonography(EUS), computed tomography (CT) and magnetic resonance cholangiopancreatography (MRCP), or elevated serum levels of ALT (> 60 U/L) and a BMI < 30 kg/m^2^ indicates an episode of acute pancreatitis with a biliary origin [3]. Hypertriglyceridemia acute pancreatitis was confirmed by triglyceride levels > 1000 mg/dL or triglyceride levels between 500 mg/dL to 1000 mg/dL together with emulsion plasma and without any other obvious causes [1, 4].

The diagnosis of local complications was performed according to the 2012 revision of the Atlanta Classification. Infected pancreatitis necrosis (IPN) could be diagnosed by the presence of extra luminal gas in the pancreatic and/or peripancreatic tissues on CECT or by a positive bacterial culture of the necrosis from the fine-needle aspiration or drainage [1]. As this study being retrospectively, one clinical feature as “high fat diet” could not be defined exactly according to the standard definition. So here, we inquired the patients or their relatives to describe the normal typical 1-day diet. By calculating the constituent ratio, we defined the “high fat diet” as fat accounts over 30–35%.

This study was performed according to the principles of the Declaration of Helsinki (modified 2000) and was approved by the institutional review board of Jinling Hospital.

### Statistical analysis

We used SPSS 18.0 statistical software package (IBM Analytics, Armonk, NY) for statistical analyses. As the following tables show, datas were presented as median plus interquartile range (IQR) for continuous variables and absolute numbers and percentages for categorical variables. The x^2^ test was used for analyzing categorical variables and Student-test or Mann–Whitney test was used for analyzing continuous variables. Statistical significance was considered as a *P* value of < 0.05 (2-tailed).

## Results

### Baseline characteristics and clinical features

**Fig. 1:**
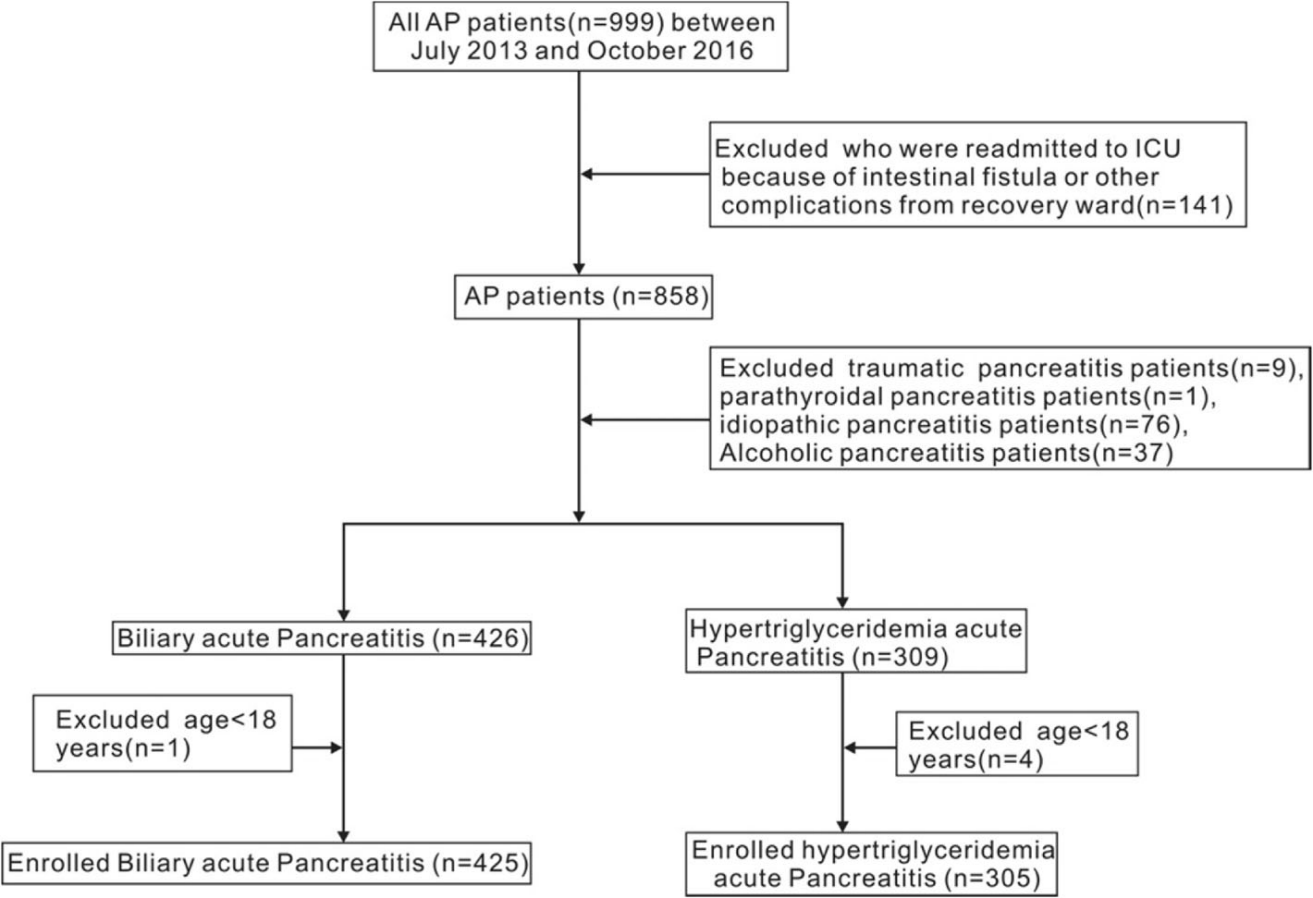
Study selection for patients

Nine hundred and ninety-nine patients were initially screened and eventually 730 patients were enrolled. The guidelines and procedures were shown in Fig. 1. Firstly, 141 patients were excluded, who were re-admitted to ICU not because of AP but other complications, such as intestinal fistula, from the recovery ward. Secondly, the patients were divided into different groups by the etiology according to the revised Atlanta criteria, which were done by two independent physicians [1]. Traumatic acute pancreatitis patients (*n* = 9), parathyroidal acute pancreatitis patients (*n* = 1), idiopathic acute pancreatitis patients (*n* = 76), alcoholic acute pancreatitis patients (*n* = 37) were also exclude. After that, we got 426 biliary acute pancreatitis (BAP) patients and 309 hypertriglyceridemia acute pancreatitis (HTG-AP) patients. Thirdly, we excluded five patients with age < 18, as 1 BAP patient and 4 HTG-AP patients. Ultimately, two groups were enrolled in this study, namely, biliary acute pancreatitis (BAP) (*n* = 425) and hypertriglyceridemia acute pancreatitis (HTG-AP) (*n* = 305) group.

The baseline characteristics of BAP and HTG-AP were displayed in Table 1. Compared to BAP patients, the HTG-AP patients were younger (40 vs 51, *p* < 0.01), with higher BMI (27 vs 22.7, *p* < 0.01), and more males (214/91 vs 242/183, *p* < 0.01), and higher incidence of diabetes (32.1% vs 12.9%, *p* < 0.01) and fatty liver (43.9% vs 15.1%, *p* < 0.01) and higher fat diet rate (42.6% vs14.6%, *p* < 0.01). Meanwhile, HTG-AP patients had significantly higher APACHEII score (*p* < 0.01) than BAP. Besides that, some similarities were also found between two groups, such as hypertension history, and duration from AP onset to transfer to our center.

**Table 1:** Demographic data and baseline characteristics of the patients

Characteristic	Biliary acute Pancreatitis (*n* = 425)	Hypertriglyceridemia acute Pancreatitis (*n* = 305)	*P* value
Age, year	51(43,64)	40(33,47)	*P* < 0.01
Gender, male/female	242/183	214/91	*P* < 0.01
BMI	22.7(20.1,25.2)	27(24.9,30.4)	*P* < 0.01
APACHE II score	8(6,12.5)	11(7,18)	*P* < 0.01
Hypertension	120(28.2%)	81(26.6%)	0.675
Diabetes mellitus	55(12.9%)	98(32.1%)	*P* < 0.01
Fatty liver	64(15.1%)	134(43.9%)	*P* < 0.01
High fat diet	62(14.6%)	130(42.6%)	*P* < 0.01
Transfer from other hospitals	406(95.5%)	295(96.7%)	0.449
Time taken for the patients transfer to our center after onset of symptoms, Days	10(4,30)	6(3,17)	0.541

*BMI* body mass index, *APACHE II* Acute Physiology and Chronic Health Evaluation II

All the patients initially received standard medical treatment according to the recent international guidelines [5]. Following a standard protocol (Additional file 1: Figure S1), fluid resuscitation was performed for each patient. The protocol includes the timing to initiation of hydration, rate of hydration and the appropriate solution. Moreover, 91 HTG-AP patients received apheresis.

**Fig. 2:**
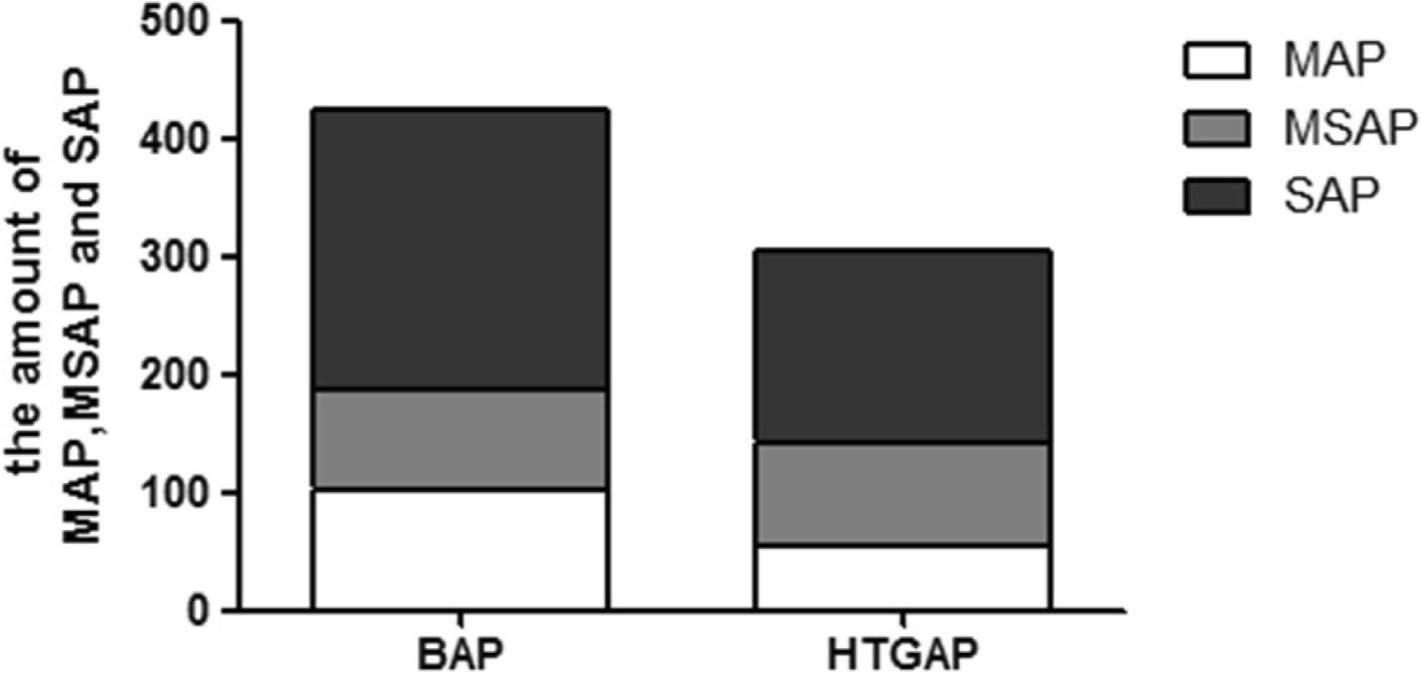
The amount of MSAP, AP, SAP in BAP and HTG-AP group

According to the modified Atlanta Criteria, the AP patients were classified into mild acute pancreatitis (MAP), mild severe acute pancreatitis (MSAP), and severe acute pancreatitis (SAP). The percentages of MAP, MSAP, SAP were respectively 24, 19.8 56.2% in BAP, and 17.7, 28.9 and 53.4% in HTG-AP (Fig. 2).

### Systemic complications and laboratory data

The laboratory data were displayed in Table 2, and systemic complications in Table 3. The results suggested that more ARDS (*p* = 0.039), AKI (*p*<0.001)and MODS(*p* = 0.032) occurred in HTG-AP, together with significantly higher CRP (*p*<0.01) and creatinine (*p* = 0.031). Also, there were more deep venous thrombosis (DVT) (27 (6.4%) vs 37 (12.1%), *p* = 0.008) in HTG-AP than BAP. However, the other data were similar in two groups, as coagulation indexes of each group, hemoglobin, blood platelet, prothrombin time, activated partial thromboplastin time, and D-Dimer. 6(1.4%) BAP patients, but no HTG-AP patient, had suffered chylous fistula.

**Table 2:** Initial Laboratory data of the patients

Physiological Indexes	Biliary acute Pancreatitis (*n* = 425)	Hypertriglyceridemia acute Pancreatitis (*n* = 305)	*P* value
Amylase	86(41,219)	69(34,159.5)	0.13
Lipase	295(107,706)	355(127.5778.5)	0.599
WBC	11(7.5,14.9)	10.4(7.7,13.6)	0.306
CRP	114(47.4173.1)	152.1(87.6210.3)	*P* < 0.01
IL-6	60(30.4135.9)	98(51.7174.1)	0.282
Procalcitonin(PCT)	0.3(0.15,1.2)	1(0.2,3.4)	0.128
Urine toxic nitrogen	4.9(3.4,7.6)	5.1(3.7,8.8)	0.07
Creatinine	55(42,72)	60(46,84)	0.031
Total bilirubin (TBil)	19.4(13.1,31.1)	19.4(13.2,29.4)	0.381
Conjugated bilirubin(DBil)	13(4,36)	13(4,28)	0.365
Alanine aminotransferase(ALT)	37(23,73)	25(19,39)	*P* < 0.01
Aspartate transaminase(AST)	30(21,52)	31(20,50)	0.408
Hemoglobin	107(91.5127)	106(85,133)	0.709
Blood platelet	194(134,269)	178(119,244.5)	0.058
Prothrombin time(PT)	13.4(12.5,14.5)	13.1(12.5,14.3)	0.58
Activated partial thromboplastin time(APTT)	34(29.9,39.7)	32(28,36.4)	0.147
D-Dimer	3.3(1.5,5.9)	3.6(2.0,7.1)	0.141

*WBC* white blood cell, *CRP* C-reactive protein

**Table 3:** Systemic complications between the patients

Variable	Biliary acute Pancreatitis (*n* = 425)	Hypertriglyceridemia acute Pancreatitis (*n* = 305)	*P* value
ARDS	130(30.6%)	116(38.0%)	0.039
AKI	91(21.4%)	105(34.4%)	*P* < 0.01
Intra-abdominal hypertension	23(5.4%)	28(9.2%)	0.056
Shock	66(15.5%)	49(16.1%)	0.838
Intra-abdominal hemorrhage	56(13.2%)	34(11.1%)	0.427
Sepsis	39(9.2%)	21(6.9%)	0.278
Portal vein thrombosis	47(11.1%)	26(8.5%)	0.317
Deep venous thrombosis	27(6.4%)	37(12.1%)	0.008
Acute hepatic injury	45(10.6%)	21(6.9%)	0.090
Gastrointestinal fistula	75(17.6%)	44(14.4%)	0.265
Digestive tract hemorrhage	11(2.6%)	8(2.6%)	1
Chylous fistula	6(1.4%)	0(0.0%)	0.044
Diarrhea	9(2.1%)	9(3.0%)	0.479
Ileus	7(1.6%)	11(3.6%)	0.144
MODS	96(22.6%)	91(29.8%)	0.032

*ARDS* acute respiratory distress syndrome, *AKI* acute kidney injury, *MODS* multiple organ dysfunction syndrome

Then, we wonder if the significantly higher incidence of ARDS, AKI and DVT in HTG-AP was affected by only the etiology or other factors, such as age, gender, body mass index, diabetic mellitus, fatty liver and high-fat diet. So, we did the multivariate analysis to determine the association of ARDS/AKI/DVT with the etiology (Table 4). On multivariate logistic regression of ARDS/AKI/DVT adjusting for etiology, age, gender, body mass index, diabetic mellitus, fatty liver and high-fat diet, HTG-AP was found to be independently associated with more AKI, and higher BMI with more AKI, ARDS and DVT.

**Table 4:** Multivariate analysis showing association of proposed risk factors with ARDS/AKI/DVT

Multivariate analysis	ARDS		AKI		DVT	
	OR(95% CI)	*P* value	OR(95% CI)	*P* value	OR(95% CI)	*P* value
Etiology	0.88(0.58,1,32)	0.52	0.62(0.40,0.96)	0.03	1.03(0.53,2.00)	0.93
Age	0.99(0.98,1.01)	0.25	0.99(0.98,1.00)	0.17	1.01(0.99,1.03)	0.36
Gender	0.93(0.67,1.29)	0.66	0.69(0.48,0.99)	0.04	1.45(0.84,2.50)	0.19
BMI	0.95(0.91,0.98)	0.004	0.96(0.92,1.00)	0.03	0.91(0.86,0.97)	0.002
Diabetes mellitus	1.38(0.92,2.07)	0.12	1.34(0.87,2.05)	0.18	0.85(0.46,1.57)	0.60
Fatty liver	0.99(0.68,1.45)	0.98	0.79(0.54,1.17)	0.24	0.67(0.38,1.19)	0.17
High fat diet	0.76(0.52,1.10)	0.14	0.88(0.59,1.31)	0.53	0.79(0.44,1.41)	0.42

*ARDS* acute respiratory distress syndrome, *AKI* acute kidney injury, *DVT* deep venous thrombosis

Then, we divided HTG-AP patients into three groups according to their TG level (peak TG level within 72 h of hospital admission with AP): group A:≤10.2 mg/dl (less than the first quartile), group B: 10.3–21.9 mg/dl (between the first and third quartiles), and group C:≥22 mg/dl (more than the third quartile). The Cochran-Armitage trend test have been done to compare characteristics among three groups, results are displayed in Additional file 2: Table S1 and Additional file 3: Figure S2. However, higher TG level was not related with the incidence of systemic complication.

### Local complications

The analysis of local complications, include acute peripancreatic fluid collection, pancreatic pseudocyst, acute necrotic collection, walled-off necrosis, and IPN, as shown in Table 5. The results in Table 5 showed that, more IPN were found in the BAP patients (193 (45.4%), 106 (34.8%), (*p* = 0.005)), although similarity in acute necrotic collection (292 (68.7%), 213 (69.8%), (*p* = 0.807)).

**Table 5:** Local complications between BAP and HTG-AP

Variable	Biliary acute Pancreatitis (*n* = 425)	Hypertriglyceridemia acute Pancreatitis (*n* = 305)	*P* value
Acute peripancreatic fluid collection	120(28.2%)	104(34.1%)	0.104
Pancreatic pseudocyst	11(2.6%)	8(2.6%)	1
Acute necrotic collection	292(68.7%)	213(69.8%)	0.807
Walled-off necrosis	5(1.2%)	3(1.0%)	1
Infected pancreatitis necrosis	193(45.4%)	106(34.8%)	0.005

### Outcome

The outcome comparison was shown in Table 6. The two groups were not statistically different in terms of in-hospital mortality. Thanks to our four-step drainage strategy [6], the amount of patients who need surgery was less than 11%.The length of hospital stay or ICU stay between two group was similar and nearly 60% needed to be admitted to ICU.

**Table 6:** Outcome comparisons between the patients

Variable	Biliary acute Pancreatitis (*n* = 425)	Hypertriglyceridemia acute Pancreatitis (*n* = 305)	*P* value
Hospital mortality, no.	36(8.5%)	24(7.9%)	0.787
Need of surgery, no.	39(9.2%)	33(10.8%)	0.529
ICU admission	260(61.2%)	186(61.0%)	1
Length of ICU stay, days	4(2,12)	4(2,10.5)	0.975
Length of hospital stay, days	9(4,22.5)	9(5,23)	0.58
Cost, Thousand CHY	48.7(22.8157.8)	51.0(27.9134.6)	0.623

*ICU* intensive care unit

## Discussion

This retrospective study demonstrated significantly different clinical features between HTG-AP and BAP patients. The results indicated that HTG-AP patients were younger, more male, more ratio of high fat diet, higher BMI and higher prevalence of AKI/ARDS/DVT/MODS, but, the BAP patients had higher incidence of IPN and chylous fistula. Moreover, the multivariate analysis showed that HTG-AP was independently associated with more AKI as adjusting by age, gender, body mass index, diabetic mellitus, fatty liver and high-fat diet, but not with ARDS and DVT. However, BMI was found to be independently associated with AKI, ARDS and DVT. The results suggested that higher incidence of AKI in HTG-AP was significantly and independently associated with the etiology, but more ARDS and DVT were more due to higher BMI.

HTG-AP is a rare but well-documented type of AP in Western countries (1.3–5%) [7, 8]. Recently, accumulating data have shown that HTG-AP has become the second common cause of AP in China, with a reported incidence up to12.3% in 2003 [9], 18.1% in 2007 [10], and 25.6% in 2013 [11], much higher than that in Western countries. In our center, which is the largest AP referral center in China, HTG-AP accounted for about 30% from 2013 to 2016, being also the second leading cause of AP. Previous studies had compared HTG-AP with other common etiologies, but the results vary. Linares et al. [12] reported that patients with HTG-AP suffered a more severe clinical course than those with alcohol or gallstone-induced based on the index such as ICU admission, CRP, and Balthazar scores and similar findings were repeatedly reported in the literature [13]. Tai et al. [14] reported no significant difference in specific complications like ARDS, AKI, gastrointestinal bleeding and sepsis. Besides, clinical studies assessing the impact of the TG level on the severity of AP also showed conflicting results. Zhang et al. [15], S. Balachandra et al. [16] and Fortson MR et al. [7] showed no difference detected in severity, based on APACHE II scores or complications in patients with the level of TG. While some reports demonstrated that patients with high level of triglyceride may suffer worse clinical outcome. A previous study showed that the level of TG in AP patients are independently and proportionally correlated with persistent organ failure regardless of etiology [17], in another study, AP patients with HTG (> 500 mg/dL) had higher 24 h APACHE II scores, more systemic complications and higher mortality [18].

The underlying pathogenesis of HTG-AP is not fully understood and the most widely accepted explanation hitherto is the “free fatty acids (FFA)” theory. It is assumed that at the onset of pancreatitis, a large amount of pancreatic lipase releases into the systemic circulation hydrolyzing serum TGs and adipose tissue, which would consequently generate high concentrations of free fatty acids with detergent properties. Free fatty acid micelle complexes injure the vascular endothelium and acinar cells of the pancreas, making internal environment increasingly acidic and ischemic, which triggers further free fatty acid toxicity and accelerate systemic inflammatory response [19]. Furthermore, direct tissue injury and lipotoxicity via mitochondrial stress [20–22] may cause the up-regulation of cytokines and the inflammatory cascade, predisposing to systemic inflammatory response [22].

In the complications of AP, the incidence of AKI was also found to be higher in HTG-AP in other reports [23]. Wu et al. indicated that TG may do a direct damage to renal parenchyma as reacting with pancreatic lipase around kidney tubules. The pancreatic enzymes assembled in glomerulus aggravates the damage of renal function [23]. Also Scheuer et al. reported that, the infiltrated TG in glomerular and tubulointerstitial could make the glomerulosclerosis grow worse in mouse model [24].

Moreover, the results in this study also showed that higher BMI was independently associated with AKI, ARDS and DVT in multivariate analysis. Up to date, quite a few articles had showed that BMI may increase the severity of AP from clinical findings and animal models [25]. A possible explanation is that adipose tissue appears as chronic inflammation releasing pro-inflammatory cytokines and obese individuals are more likely to have lifestyle-related chronic diseases/ respiratory problems [25, 26]. Obesity has also been inferred associated with an increased risk of extrapancreatic complications such as shock, renal failure, respiratory insufficiency, and fatal outcome [27, 28].

Furthermore, the results found the BAP patients suffered more infected local complications than HTG-AP. In BAP patients, when the gallstones pass through the Vater ampulla, spasm, fibrosis and obstruction of the hepatopancreatic ampulla happens, resulting in biliopancreatic reflux and the exclusion of bile and pancreatic juices. Then the elevated levels of bile and pancreatic juices and activation of pancreatic enzymes are responsible for pancreatitis attacking ultimately in this abnormal physiological status [3]. The refluxing of infected bile transfer into the pancreatic duct may generate *Escherichia coli* and other bacteria leading to the infection [29].

## Conclusions

In conclusion, this study showed significantly different clinical features between HTG-AP and BAP patients. HTG-AP patients suffered higher occurrence rates of AKI/ARDS/DVT/MODS, while IPN was more common in BAP patients. After the multivariate analysis adjusted by age, gender, BMI, diabetes, fatty liver and high fat diet, HTG-AP was found to be significantly and independently related with more incidence of AKI. However, higher BMI was found to be related with AKI, ARDS and DVT. Further, larger prospective studies should be performed to study the features between the different etiologies of AP and the effect of BMI on disease severity.

## Additional files

Additional file 1: **Figure S1.** The standardized protocol for the fluid resuscitation treatment. (DOCX 213 kb)

Additional file 2: **Table S1.** Characteristics in three groups divided by triglyceride levels. (DOCX 15 kb)

Additional file 3: **FigureS2.** Proportion of systemic complication with three groups according to the value of TG level in patients with HTG-AP using the Cochran-Armitage trend test. A. Proportion of ARDS with three groups according to the value of TG level in patients with HTG-AP. B. Proportion of AKI with three groups according to the value of TG level in patients with HTG-AP. C. Proportion of DVT with three groups according to the value of TG level in patients with HTG-AP. Cochran-Armitage test for trend was analyzed. (DOCX 26 kb)

## Abbreviations

AKI: Acute kidney injury
ALT: Alanine aminotransferase
AP: Acute pancreatitis
APACHE II: Acute physiology and chronic health evaluation II
ARDS: Acute respiratory distress syndrome
BAP: Biliary acute pancreatitis
BMI: Body mass Index
CECT: Contrast-enhanced computed tomography
CRP: C-reactive protein
DVT: Deep vein thrombosis
EUS: Endoscopic ultrasonography
FFA: Free fatty acids
HTG: Hypertriglyceridemia
HTG-AP: Hypertriglyceridemia acute pancreatitis
ICU: Intensive Care Unit
IPN: Infected pancreatitis necrosis
IQR: Interquartile range
MAP: Mild acute pancreatitis;
MODS: Multiple organ dysfunction syndrome
MRCP: Magnetic resonance cholangiopancreatography
MRI: Magnetic resonance imaging
MSAP: Moderate severe acute pancreatitis
SAP: Severe acute pancreatitis.
SICU: Surgical Intensive Care Unit
