# Supplementary figures and images for: Significantly different clinical features between hypertriglyceridemia and biliary acute pancreatitis: a retrospective study of 730 patients from a tertiary center
Source: BMC Gastroenterol. 2018 Jun 19;18:89. doi: 10.1186/s12876-018-0821-z (PMC6007076; doi:10.1186/s12876-018-0821-z)

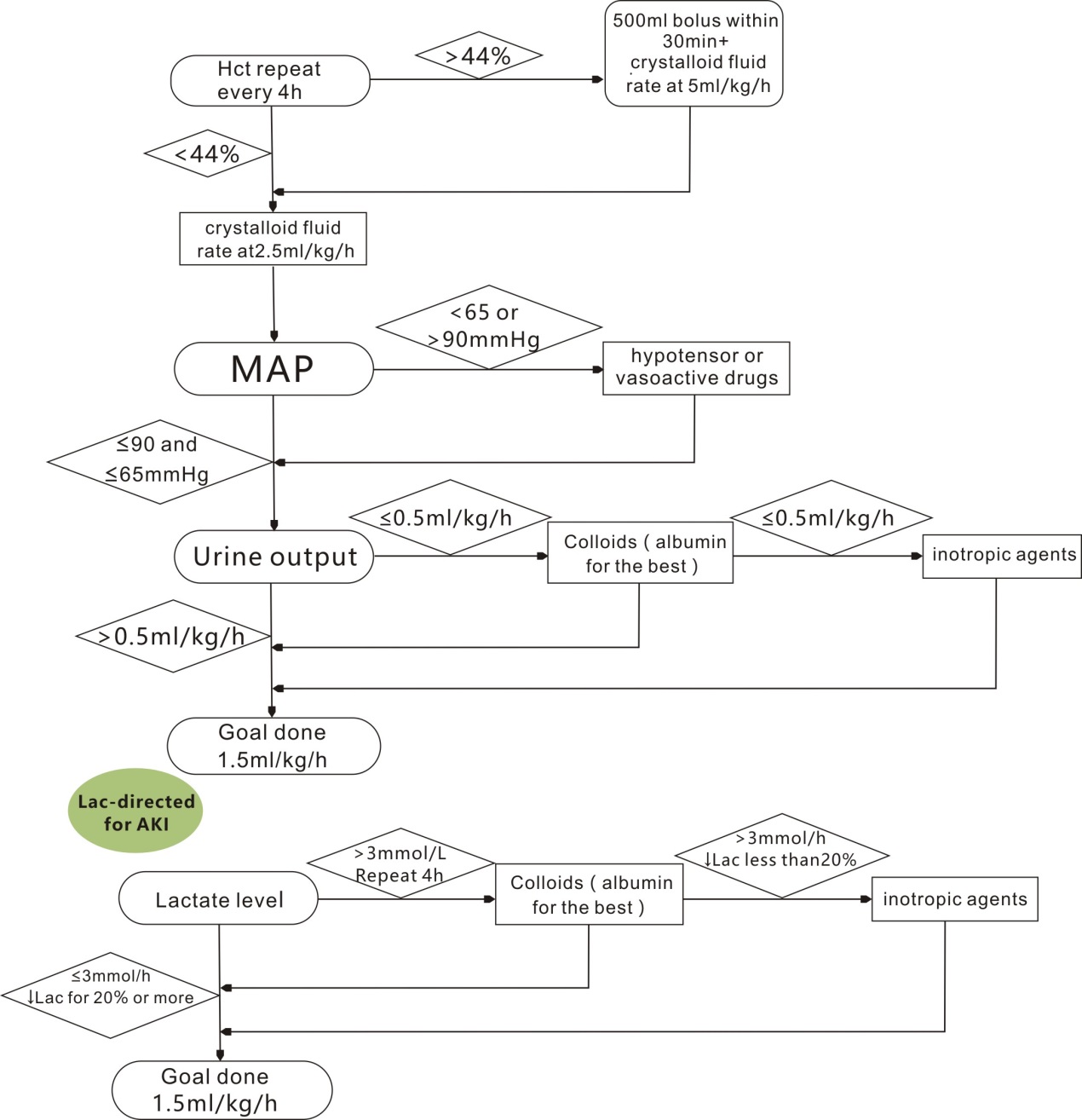

Supplement: Supplementary file 1 — Figure S1. The standardized protocol for the fluid resuscitation treatment. (DOCX 213 kb) [file 12876_2018_821_MOESM1_ESM.docx]

A.


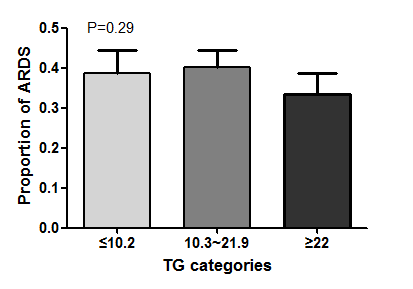


B.


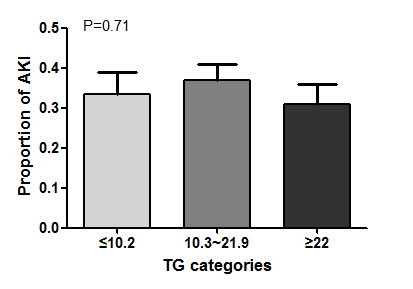


C.


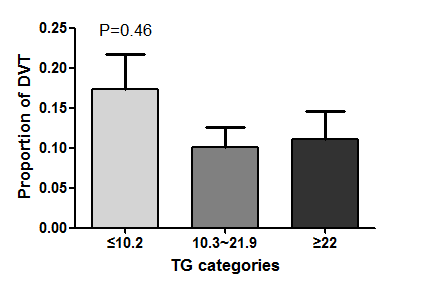

Supplement: Supplementary file 3 — FigureS2. Proportion of systemic complication with three groups according to the value of TG level in patients with HTG-AP using the Cochran-Armitage trend test. A. Proportion of ARDS with three groups according to the value of TG level in patients with HTG-AP. B. Proportion of AKI with three groups according to the value of TG level in patients with HTG-AP. C. Proportion of DVT with three groups according to the value of TG level in patients with HTG-AP. Cochran-Armitage test for trend was analyzed. (DOCX 26 kb) [file 12876_2018_821_MOESM3_ESM.docx]
