# Supplementary material for: Significantly different clinical features between hypertriglyceridemia and biliary acute pancreatitis: a retrospective study of 730 patients from a tertiary center
Source: BMC Gastroenterol. 2018 Jun 19;18:89. doi: 10.1186/s12876-018-0821-z (PMC6007076; doi:10.1186/s12876-018-0821-z)
Supplement: Supplementary file 2 — Table S1. Characteristics in three groups divided by triglyceride levels. (DOCX 15 kb) [file 12876_2018_821_MOESM2_ESM.docx]

| Characteristic | All（n=305） | Triglyceride Tertile（mg/dL） | | |
| --- | --- | --- | --- | --- |
|  |  | ≤first quartile | first ~third quartiles | ≥third quartile |
|  |  | ≤10.2（n=75） | 10.3~21.9（n=149） | ≥22（n=81） |
| Age, year | 40(33,47) | 42（34,47） | 40（33,47） | 39（32,44.5） |
| Gender, male/female | 214/91 | 58/17 | 104/45 | 52/29 |
| BMI | 27(24.9,30.4) | 26.2（24.7,29.6） | 27（25.3,32） | 27.1（24,29.6） |
| APACHE Ⅱ score | 11(7,18) | 12（6.18） | 11（7,19） | 11（6.16） |
| Hypertension | 81(26.6%) | 21(28.0%) | 44(29.5%) | 16(19.8%) |
| Diabetes mellitus | 98(32.1%) | 24(32.0%) | 45(30.2%) | 29(35.8%) |
| Fatty liver | 134(43.9%) | 42(56.0%) | 61(40.9%) | 31(38.3%) |
| High fat diet | 130(42.6%) | 32(42.7%) | 67(45.0%) | 31(38.3%) |
| Transfer from other hospitals | 295(96.7%) | 73(97.3%) | 145(97.3%) | 77(95.1%) |
| Time taken for the patients transfer to our center after onset of symptoms，Days | 6(3,17) | 4（3.10） | 6（3,16.5） | 7（3,23） |
| No. of ARDS | 116(38.0%) | 29（38.7%） | 60（40.3%） | 27（33.3%） |
| No. of AKI | 105(34.4%) | 25（33.3%） | 55（36.9%） | 25（20.9%） |
| No. of DVT | 37(12.1%) | 13（17.3%） | 15（10.1%） | 9（11.1%） |
